# Male increase in brain gene expression variability is linked to genetic risk for schizophrenia

**DOI:** 10.1038/s41398-018-0200-0

**Published:** 2018-08-01

**Authors:** Junfang Chen, Han Cao, Andreas Meyer-Lindenberg, Emanuel Schwarz

**Affiliations:** Medical Faculty Mannheim, Department of Psychiatry and Psychotherapy, Central Institute of Mental Health, Heidelberg University, Mannheim, Germany

## Abstract

Schizophrenia shows substantial sex differences in age of onset, course, and treatment response, but the biological basis of these effects is incompletely understood. Here we show that during human development, males show a regionally specific decrease in brain expression similarity compared to females. The genes modulating this effect were significantly co-expressed with schizophrenia risk genes during prefrontal cortex brain development in the fetal period as well as during early adolescence. This suggests a genetic contribution to a mechanism through which developmental abnormalities manifest with psychosis during adolescence. It further supports sex differences in brain expression variability as a factor underlying the well-established sex differences in schizophrenia.

## Introduction

Schizophrenia is a severe developmental mental illness with an incidence approximately 1.4 times higher in men compared to women^1^. The disorder is substantially heritable and a large number of common and rare variants have been associated with illness risk^2–5^. A widely accepted neurodevelopmental hypothesis posits that genetically determined alterations in early brain development interact with developmental changes during adolescence in the prefrontal cortex to lead to the manifestation of psychosis^6,7^. Consistent with this, developmentally changing prefrontal cortex expression has been found to be linked to neuronal differentiation and maturation, as well as genetic schizophrenia risk^8^.

In men, the illness has a more severe course characterized by more pronounced negative symptoms as well as cognitive impairment^9,10^, although evidence has been reported that substance abuse in men may confound such clinical differences^11^. Males with schizophrenia have also, albeit inconsistently, been reported to have a lower age of onset, show more pronounced alterations of brain morphology, and poorer response to antipsychotic medication^9,11–13^. Genetic risk associations, as well as molecular profiles, contain sex-dependent factors^14,15^ and sex hormones are thought to play an important role for illness course^9,16^, but again little is known about the underlying neurobiological mechanisms.

We pursued a novel strategy to explore how biological sex differences may impact on the manifestation of genetic risk and the clinical sex differences of schizophrenia. Inspired by a recent study on the human brain connectome^17^, we tested whether during development human brain gene expression is more variable in males than females. We hypothesized that such increased expression variability might contribute to a predisposition of males for heritable neurodevelopmental disorders. A similar hypothesis has previously been explored for HIV, where gene expression variability has been suggested as a modulator for susceptibility to infection^18^. Our study is further motivated by previous identification of sexual dimorphisms of brain expression^19–21^, protein abundance^22^, as well as genetic and epigenetic factors modulating gene expression noise^23,24^, supporting the possibility of links between polygenic risk and expression variance. The longitudinal exploration of variability differences is further motivated by previous identification of differential variance of transcriptional regulators during human embryonic development^25^. Analysis of gene expression variability has also been successfully applied to identify genes and pathways implicated in several illnesses and highlighted such variability as an informative biological signal^26,27^.

Expression variability as genetic risk mediator can capture polygenic effects beyond sex differences of expression. To investigate this, we identified genes driving brain region and age-specific variability differences between sexes and tested whether these were associated with expression of schizophrenia risk genes.

## Materials and methods

### Data preprocessing

To characterize brain expression throughout the human lifespan, we used data from the BrainSpan: Atlas of the Developing Human Brain (funded by ARRA Awards 1RC2MH089921-01, 1RC2MH090047-01, and 1RC2MH089929-01 and available from: http://developinghumanbrain.org), as well as Braincloud microarray data (GSE30272^28^, available from the GEO database^29^).

The primary analysis was performed on BrainSpan exon microarray data (GSE25219, preprocessed as described in ref. ^30^) due to availability of a larger sample number. BrainSpan RNA sequencing (RNAseq) data was used for replication and Braincloud data for validation of findings. BrainSpan data comprised transcriptome-wide expression information on subjects between the 6th post-conceptional week (PCW) and 40 years of age (Table 1, Supplementary Tables 2, 8, and 9). We did not consider older subjects, as sex effects on risk are not likely to manifest beyond the typical age of onset that ranges between late adolescence and early adulthood. As performed by Willsey et al.^31^, subjects were grouped in age-bins by a windowing approach that joins three consecutive age periods into a single group.

**Table 1:** BrainSpan exon microarray sample numbers for males and females across 11 age-bins and 4 brain regional clusters after data preprocessing

		Males				Females			
Age-bin	Regional cluster	1	2	3	4	1	2	3	4
1		12 (3)	21 (4)	11 (4)	1 (1)	20 (4)	24 (4)	12 (4)	7 (4)
2		23 (5)	31 (6)	16 (6)	5 (4)	20 (4)	24 (4)	12 (4)	7 (4)
3		23 (5)	27 (5)	14 (5)	5 (4)	33 (7)	39 (7)	20 (7)	13 (7)
4		23 (5)	27 (5)	14 (5)	8 (5)	27 (6)	31 (6)	16 (6)	12 (6)
5		15 (3)	18 (3)	9 (3)	6 (3)	27 (6)	31 (6)	16 (6)	12 (6)
6		30 (6)	36 (6)	18 (6)	12 (6)	14 (3)	16 (3)	8 (3)	6 (3)
7		24 (5)	29 (5)	12 (4)	10 (5)	5 (1)	6 (1)	3 (1)	2 (1)
8		24 (5)	28 (5)	12 (4)	10 (5)	10 (2)	9 (2)	6 (2)	4 (2)
9		19 (4)	20 (4)	8 (3)	7 (4)	15 (3)	15 (3)	8 (3)	5 (3)
10		20 (4)	21 (4)	10 (4)	7 (4)	20 (4)	21 (4)	11 (4)	7 (4)
11		36 (8)	42 (8)	19 (7)	14 (8)	33 (7)	39 (7)	19 (7)	12 (7)

1: V1C-STC, 2: PFC-MSC, 3: STR-HIP-AMY, 4: MD-CBC (see Supplementary Table 1 for details). Subject numbers are shown in brackets

Preprocessing of all datasets followed a similar sequence of steps (Supplementary Fig. 1). Procedures performed on all datasets comprised: RNA Integrity Number (RIN) filtering (for BrainSpan exon microarray data, all donors were removed that had more than 25% of microarray samples with RIN < 7.5, as in ref. ^30^; for BrainSpan RNAseq data and Braicloud data, a more stringent filtering was performed by removing all samples with RIN < = 7.5); removal of subjects >40 years; log2 transformation of data; extraction of autosomal genes (without minimum expression filter); quantile normalization; surrogate variable determination; covariate adjustment; and outlier detection. This data contained the respective median values if multiple replicates per subject were present. Following a previously described pipeline^20^, processing of RNAseq data included two additional steps: gene-level reads per kilobase million mapped reads (RPKM) were normalized for GC content using conditional quantile normalization based on the R library *cqn*^32^ and all genes with less than 1 RPKM in more than 50% of male or female samples were removed. Surrogate variable analysis was performed to account for the potential effects of unobserved confounders^33^. The number of surrogate variables were automatically determined using the *num.sv* function of the R package *sva*^33^, using the approximation method by Leek^33,34^. The underlying full model matrix contained gender, whose effects on expression variability should be preserved, as well as age, PMI, RIN, and brain pH (as well as an array indicator for Braincloud data). The null-model matrix contained all covariates but gender. Age was used as a covariate, to prevent artifactual correlations between genes due to their joint association with age. This is particularly important for age-bins covering a broader range of ages, where significant correlations between age and expression can be expected. The number of surrogate variables determined for BrainSpan exon microarray was 0, 2 for BrainSpan RNAseq and 0 for the Braincloud data. Covariate adjustment was performed via residualization against all covariates described above (except for gender) using linear models. Missing brain pH values were replaced by the mean of non-missing values.

### Outlier detection

After preprocessing, principal component analysis was used to exclude outliers (Supplementary Fig. 2). For this, we identified separately for males and females observations that deviated more than 3 standard deviations from the mean of the respective first two principal components. This removed 7 samples in the BrainSpan exon microarray data (6 from male donors), 11 observations in the BrainSpan RNAseq data (6 from male donors), and 1 outlier (from a female donor) in the Braincloud data.

### Schizophrenia risk genes

Schizophrenia risk variants, loci, and associated genes were taken from ref. ^5^ (Supplementary Table 3). Previous analyses have pursued different approaches to identify genes linked to genetic schizophrenia risk. Among these approaches is the selection of all genes or those within a certain distance from a given locus^5^, or genes affected by index variant eQTLs^35^. For the present study, we aimed to identify a single gene per locus. This was due to the risk of introducing statistical bias from including multiple genes per locus, caused by (1) the undue influence of loci harboring a larger number of genes and (2) the gene–gene correlation of genes in close chromosomal proximity. Therefore, for loci harboring multiple genes, we here used the gene in closest chromosomal proximity to the genome-wide significant index variant. If a locus contained more than one index variant, we selected the gene in closest chromosomal proximity to the most significant index variant. Chromosomal locations were determined from the R library org.Hs.eg.db., vs. 3.1.2 (genome build hg19, assembly GRCh37). Genes within the MHC region were not considered due to their significant linkage disequilibrium pattern. Two loci mapped to the genes IMMP2L and TCF4, and these were considered only once for subsequent analyses. C10orf32, C12orf79, and VPS14C were not annotated by the library org.Hs.eg.db. and not considered for further analysis. The final set of schizophrenia risk genes contained 100 genes, of which 97 were autosomal. Of these, 87 were part of the BrainSpan dataset (see Supplementary Table 3).

### Analysis of expression similarity

First, all samples were identified for a given brain regional cluster and age-bin. Based on such data subset, we performed a three stage resampling approach separately for males and females. The objective of this resampling was to quantify the expression similarity (and its confidence interval) between subjects while accounting for the non-independence of multiple samples taken from the same donor:

1. First, we randomly selected a single sample per subject to prevent an impact of sample non-independence on results.
2. Second, we took a bootstrap sample of subjects by sampling with replacement and chose the unique set of subjects. This was performed to prevent the perfect correlation between multiply selected samples.
3. Finally, we subsampled the selected subjects, such that the same number of subjects was chosen for males and females. This was aimed at preventing an influence of unequal sample numbers on results.

Then separately for males and females, we determined the pairwise Pearson correlation coefficients between all subject pairs using expression values from all genes. The mean of these estimates was used as an estimate of expression similarity between subjects for a given regional cluster age-bin combination. Only the upper triangular matrix of a given correlation matrix was used for estimation. This entire resampling was repeated 100 times and the mean value (for confidence intervals the upper and lower 2.5% percentile) of obtained estimates used to quantify expression similarity.

The difference between males and females was then quantified as the mean difference between the point estimates of each regional cluster age-bin combination. To assess significance, the resampling procedure was repeated 1000 times. During each repetition, gender information was permuted for a given regional cluster age-bin combination, such that different samples of the same subject were always assigned the same gender. The frequency of bootstrapping point estimates at least as high as the one obtained from non-permuted data was used as empirical *P*-value and corrected for multiple comparisons according to the method of Bonferroni. To perform two-sided tests, absolute values were used for this calculation.

### Identification of genes driving expression similarity differences

We anticipated that genes driving the difference of expression similarity between males and females would likely show strong differences in expression variance between sexes. For each regional cluster age-bin combination, we therefore performed the same resampling strategy as described above. For a given set of subjects (males and females separately), we then determined the standard deviation of expression for a given gene. These estimates were averaged over 100 resampling repetitions. We then determined the ratio of these averages between males and females and used the 100 genes (arbitrary cut-off) with the highest ratio as “variability genes”. To test whether these gene sets were also “variability genes” in replication (BrainSpan RNAseq data) and validation (Braincloud) data, we determined the difference of expression similarity estimates (using the resampling strategy described above) between males and females. An empirical *P*-value was then determined by comparing this estimate against those derived from random “variability genes” identified as described below (1000-fold resampling, one-sided test).

### Testing associations with schizophrenia risk genes

To explore associations between variability genes and schizophrenia susceptibility genes, the co-expression between the two gene sets was determined for a given regional cluster age-bin combination, by calculating a matrix of all pairwise Pearson correlation coefficients using expression values from both gene sets. The median value of this correlation matrix was then used as a measure of co-expression. Again, these calculations were determined as part of the resampling procedure described above, with the exception of the third step (undersampling to obtain equal numbers of male and female subjects), since calculations were performed using males only.

Significance was determined using 1000 fold resampling. During each repetition and for each regional cluster age-bin combination, the low number of donors prevented meaningful permutation of gender information. Therefore, random “variability genes” were selected such that for each real variability gene, one gene with a standard deviation of expression within 5% of the original gene was randomly chosen. The resulting co-expression values were then used to form null-distributions. Empirical *P*-values were determined as the frequency of co-expression values at least as high as that observed from real data (one-sided test). Since a total of 22 sets of variability genes were tested, *P*-values were corrected for the Family Wise Error Rate according to the method of Bonferroni.

### Analysis of schizophrenia specificity

To test the specificity of the co-expression between variability genes and schizophrenia susceptibility genes, five additional analyses were performed, using different selections of “susceptibility genes”: (I) Random selection of schizophrenia susceptibility genes for a given locus (instead of based on physical proximity to the index SNP). (II) Random selection of genes from loci with comparable DNA sequence variability compared to the schizophrenia loci. For this analysis, the number of common (MAF > = 1%) variants recorded in dbSNP (GRCh37, available from https://genome.ucsc.edu/) was used as a proxy for DNA sequence variability. For each schizophrenia locus, a locus of the same size was selected from the same chromosome and retained if the DNA sequence variability was within 10% of the original locus. A random gene was then selected from the locus, extended by 20 kbp, using the R library *biomaRt*^36^. (III) Random selection of genes from the same chromosome as a given schizophrenia gene, irrespective of DNA sequence variability. (IV) Selection of genes in proximity to SNPs associated with major depressive disorder (35 genes; closest gene selected to a given index SNP, as described in ref. ^37^). (V) Selection of genes in proximity to SNPs associated with a non-psychiatric phenotype (coronary artery disease; 35 genes; random gene selected from a given susceptibility locus, as described in ref. ^38^).

### Exploratory age-windowing

To perform a “fine-mapping” of effects within a set of age-bins, we performed separate analyses for subjects within a given age-window (Supplementary Table 7). The width of the window was determined as four consecutive age entries among the recorded ages in weeks. Differences of expression similarity and co-expression with schizophrenia susceptibility genes were determined separately for each age-window as described above. Genes identified as “variability genes” of the investigated age-bins were combined and used for this analysis.

### Functional analysis

To explore biological functions of genes contributing to differences of expression similarity between sexes, we used the DAVID functional annotation tool using default settings (https://david.ncifcrf.gov/home.jsp)^39^. In this tool, enrichment is quantified based on a modified Fisher’s exact test. The 14,702 autosomal genes part of the BrainSpan exon microarray data were used as background for functional analysis. We retained all functional annotation clusters with at least one annotation term passing the False Discovery Rate corrected *P*-value threshold of 0.05.

### Code availability

Code is available from the corresponding author upon request.

## Results

### Expression similarity differences in BrainSpan exon microarray data

**Fig. 1:**
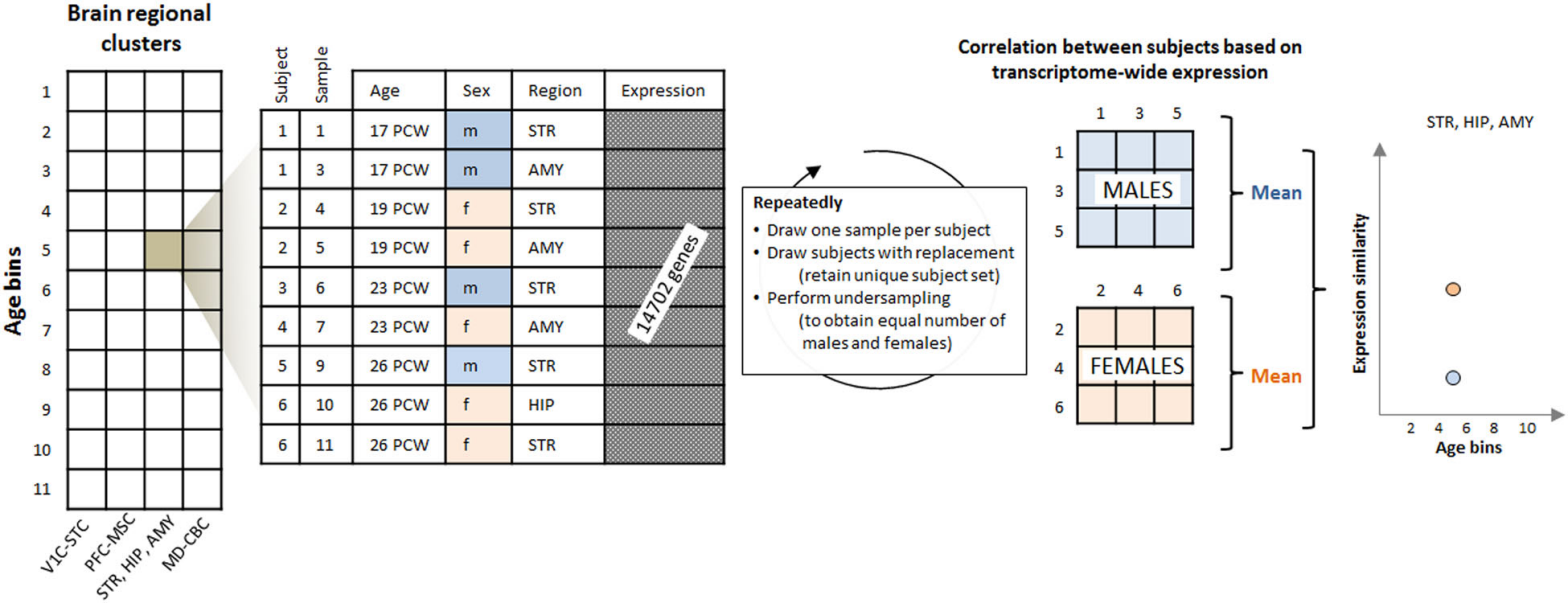
Analysis workflow. Transcriptome-wide expression data were extracted from the BrainSpan Atlas of the Developing Human Brain for each age-bin brain regional cluster combination. Age-bins and regional clusters were taken from ref. ^31^. Using a resampling procedure, expression variability was then quantified in males and females as the mean of the pairwise correlations of transcriptome-wide expression between samples from the respective subjects. PCW post conceptional week, V1C primary visual cortex, ITC inferior temporal cortex, IPC posterior inferior parietal cortex, A1C primary auditory cortex, STC superior temporal cortex, M1C primary motor cortex, S1C primary somatosensory cortex, VFC ventral prefrontal cortex, MFC medial prefrontal cortex, DFC dorsal prefrontal cortex, OFC orbital prefrontal cortex, STR striatum, HIP hippocampal anlage/hippocampus, AMY amygdala, MD mediodorsal nucleus of the thalamus, CBC cerebellar cortex

The filtered dataset contained autosomal, transcriptome-wide expression data on healthy subjects between the 6th PCW and 40 years of age^19^ (42 donors, 23 males, 14,702 autosomal genes; Fig. 1). We tested whether gender was confounded by ethnicity, but found no association (*P* = 0.77, Chi-squared test). Subjects were binned into 11 age groups and the 16 brain areas were aggregated into 4 regional clusters with similar expression values (Supplementary Tables 1 and 2, regional clustering was taken from ref. ^31^ and based on hierarchical clustering of fetal transcriptome profiles; for abbreviations, see Fig. 1): (1) the V1C-STC cluster; (2) the prefrontal and primary motor-somatosensory cortex or PFC-MSC cluster; (3) the STR-HIP-AMY cluster; and (4) the MD-CBC cluster.

**Fig. 2:**
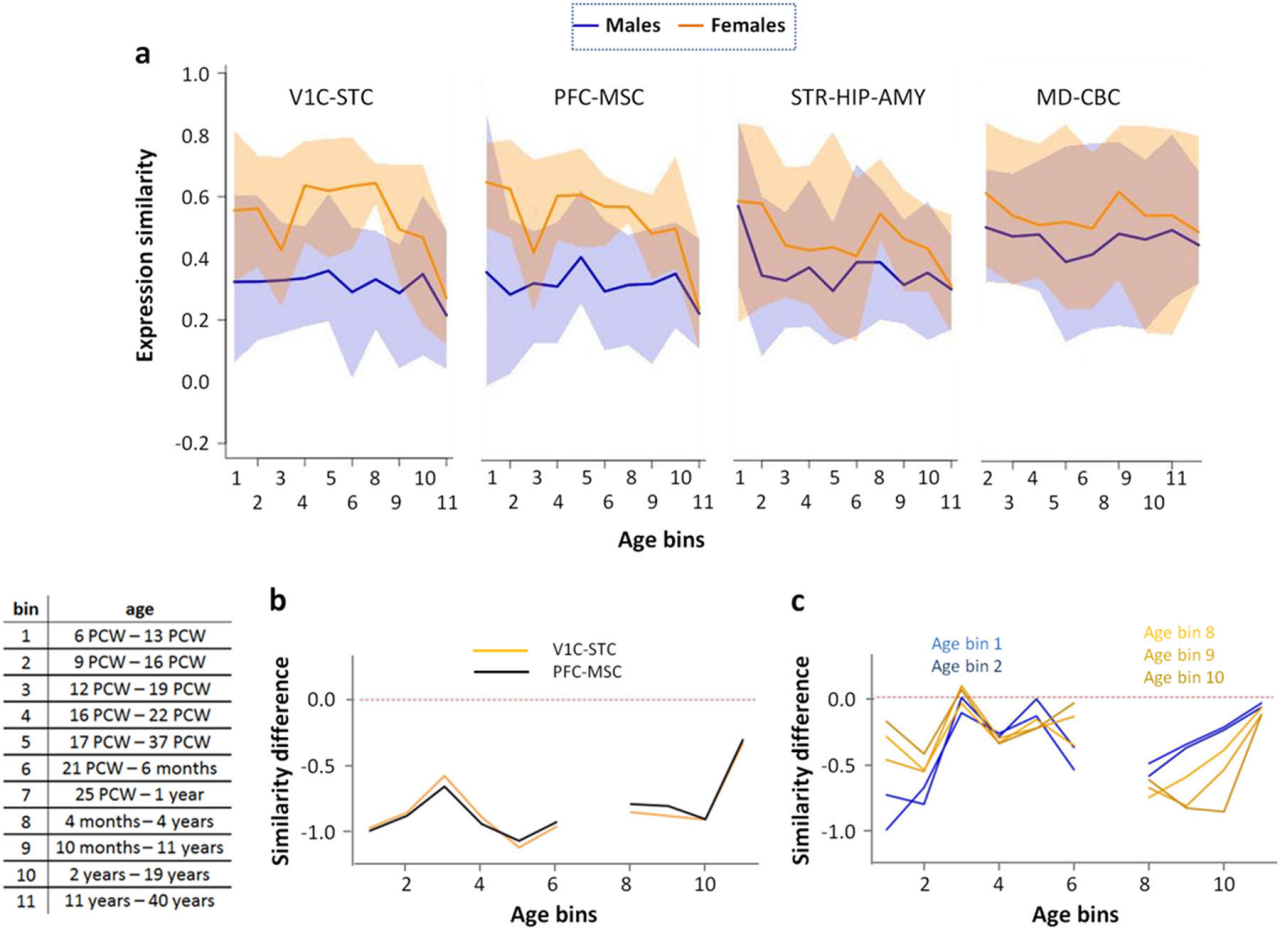
Sex differences in expression similarity in BrainSpan exon microarray data. **a** Expression similarity for four brain regional clusters: V1C-STC, PFC-MSC, STR-HIP-AMY, and MD-CBC for males (blue) and females (orange). The panels display mean estimates (solid lines) and 95% confidence intervals (shaded areas). The panels show no values for regional cluster age-bin combinations containing data from only one donor. **b** Expression variability for “variability genes”, identified separately for each given age-bin. In age-bin 7, data from only one donor was available for females. **c** Expression variability profiles for variability genes derived from age-bins 9 (10 months–11 years) and 10 (2 years–19 years) in the PFC-MSC cluster. This panel shows variability profiles for male subjects only

Figure 2a shows that despite substantial variability, males had significantly lower expression similarity compared to females in three of the four brain regional clusters (*P*_V1C-STC_ < 0.004, *P*_PFC-MSC_ < 0.004, *P*_STR-HIP-AMY_ = 0.003, *P*_MD-CBC_ = 0.080; FWER corrected). Due to the more pronounced differences in the regional clusters V1C-STC and PFC-MSC, subsequent analyses focused on these areas. Figure 2a further shows that in females, expression similarity tended to decrease across developmental time points, suggesting that inter-subject similarity was lower in adulthood compared to younger age. We aimed to explore whether sex differences in expression similarity were associated with genetic schizophrenia risk, to pinpoint a potential biological mechanism for the well-known sex differences of the disorder.

### Identification of genes driving sex differences in expression similarity

For each age-bin regional cluster combination we identified the 100 “variability genes” with the greatest ratio (male divided by female) of standard deviations of expression (see Supplementary Fig. 3 and Supplementary Dataset for a list of all “variability genes”). Figure 2b shows that expression similarity determined from these genes differed strongly between sexes.

### Co-expression between variability and schizophrenia susceptibility genes

**Fig. 3:**
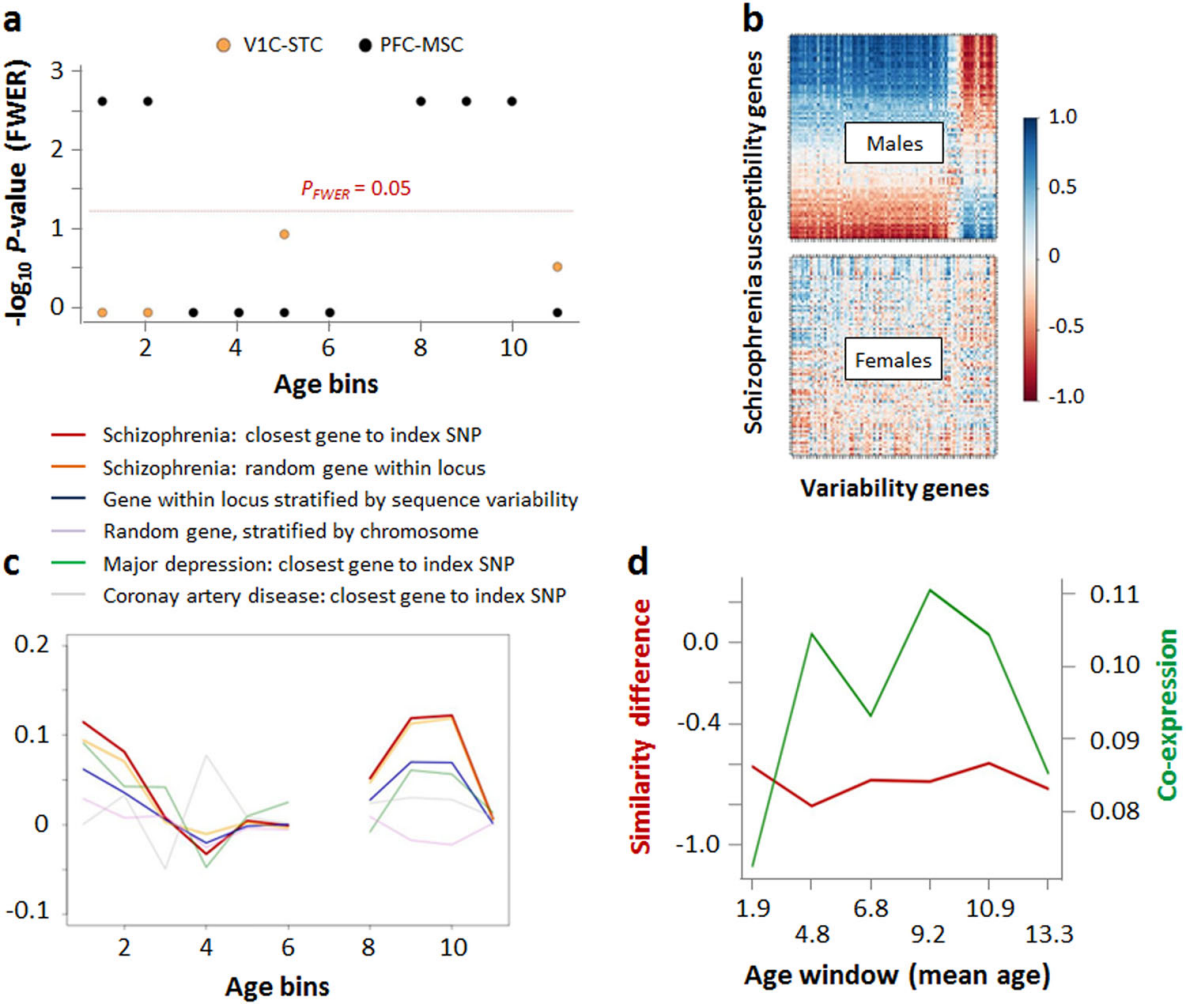
Co-expression between variability genes and schizophrenia susceptibility genes. **a** Significance of median co-expression for variability genes determined for each age-bin in the regional clusters V1C-STC, PFC-MSC, and STR-HIP-AMY of male subjects. **b** Co-expression in PFC-MSC cluster, age-bin 10, for males and females, respectively. Rows and columns were ordered separately based on median co-expression. **c** Comparison of co-expression between variability genes and schizophrenia susceptibility genes chosen based on physical proximity to index SNPs (red), random selection within a given susceptibility locus (orange), randomly selected loci with comparable DNA sequence variability compared to schizophrenia loci (blue), random genes selected from the same chromosomes as schizophrenia susceptibility genes (purple), major depression susceptibility genes (green), and genes linked to a non-psychiatric phenotype (coronary artery disease, gray). **d** Windowing of age-bins 8, 9, and 10 in the PFC-MSC cluster. The panel shows variability difference and co-expression for variability genes determined for age-bins 8–10. Co-expression was determined for males only

Next, we investigated potential relationships between these variability genes and genes harbored by the 108 well-established schizophrenia susceptibility loci^5^. This analysis was performed in males, since the lack of variance in female expression levels would prevent meaningful association analyses. Across 22 sets of variability genes (11 age-bins in the 2 regional clusters V1C-STC and PFC-MSC), we found that variability genes derived from both clusters were significantly co-expressed with schizophrenia susceptibility genes in age-bins 8 (4 months–4 years, rho_V1C-STC_ = 0.05, rho_PFC-MSC_ = 0.05), 9 (10 months–11 years, rho_V1C-STC_ = 0.10, rho_PFC-MSC_ = 0.12), and 10 (2 years–19 years, rho_V1C-STC_ = 0.07, rho_PFC-MSC_ = 0.13; all *P*_*FWER*_ < 0.022, Fig. 3a, b). Significant co-expression was additionally observed for the PFC-MSC in age-bin 1 (6 PCW–13 PCW, rho = 0.11) and 2 (9 PCW–16 PCW, rho = 0.08; all *P*_*FWER*_ < 0.022).

### Age-bin specificity and pathway analysis

Next, we explored whether differences in expression similarity were age-bin specific. Figure 2c shows that PFC-MSC variability genes of age-bin blocks 1–2 and 8–9–10 were also associated, albeit to a lesser extent, with decreased male expression similarity in the respectively other age-bin blocks.

In this brain regional cluster, the 257 genes of age-bins 8–10 were significantly linked to synaptic processes and (calcium-)ion signaling (Supplementary Table 4). Notably, the 138 variability genes from age-bins 1 and 2 in the PFC-MSC cluster were associated with similar ontological categories, including “post-synaptic membrane” and “synapse” (Supplementary Table 5). Interestingly, the genes from age-bins 1–2 and age-bins 8–10 showed only a minimal overlap (8 genes shared). These ontological associations showed regional specificity for the PFC-MSC cluster, as the V1C-STC variability genes (age-bins 8–10) that also showed significant co-expression with susceptibility genes were not associated with similar ontological categories (Supplementary Table 6). Furthermore, the ontological overlap between age-bins 1–2 and age-bins 8–10 in the PFC-MSC cluster is consistent with the correlation of the male expression similarity profiles (Fig. 2c).

### Schizophrenia specificity

To explore the specificity of co-expression results for schizophrenia, analysis was repeated using (I) schizophrenia susceptibility genes randomly selected for a given locus (instead of based on physical proximity to the index SNP), (II) genes randomly selected from loci with comparable DNA sequence variability compared to the schizophrenia loci, (III) genes randomly selected from the same chromosome as a given schizophrenia gene, irrespective of DNA sequence variability, (IV) genes in proximity to SNPs associated with major depressive disorder, (V) genes in proximity to SNPs associated with a non-psychiatric phenotype (coronary artery disease). Figure 3c shows that random and proximity-based selection of genes from schizophrenia loci yielded similar results. Despite a similar co-expression profile across age-bins, schizophrenia gene co-expression showed specificity against DNA sequence variability-stratified gene selection in age-bins 8 and 9 (*P* = 0.05) and a trend toward specificity in age-bins 2 and 10 (*P* = 0.06). Randomly selected genes (procedure III) showed substantially lower mean co-expression, leading to specificity of schizophrenia results (age-bins 8–10, *P* ≤ 0.05; age-bin 2, *P* = 0.06). For both random selection procedures, schizophrenia specificity could not be observed in age-bin 1 (*P* = 0.12 and *P* = 0.11, for procedures II and III, respectively). Genes in the proximity of SNPs linked to major depression or grip strength led to lower co-expression values in age-bins 2 and 8–10; in age-bin 1, major depression genes showed higher co-expression than the schizophrenia genes.

### Age-windowing

Finally, since age-bins 8–10 covered a broad age range (4 months–19 years), we performed an exploratory “fine-mapping” of PFC-MSC effects using an age-windowing approach. While based on small sample numbers, this analysis suggested that co-expression had a broad plateau from a mean age of 4.8–10.9 years (Fig. 3d). Differences in expression similarity between sexes were consistent across all age windows (Fig. 3d).

### Replication in BrainSpan RNAseq data

Preprocessed BrainSpan RNAseq data comprised expression information on 11,514 autosomal genes in 400 samples (37 subjects, 20 males). The transcriptome-wide expression similarity showed similar profiles as observed for exon microarray data (Supplementary Fig. 4a). Similarly, the variability genes identified from exon microarray data were also variability genes in RNAseq data (Supplementary Fig. 4b, *P* < 0.001). These genes were significantly correlated with schizophrenia susceptibility genes in the PFC-MSC regional cluster for age-bins 2 (rho = 0.01, *P* < 0.001), 9 (rho = 0.05, *P* < 0.001), and 10 (rho = 0.03, *P* < 0.001), validating exon microarray observations. For the V1C-STC cluster, we found significant associations for age-bins 3 (rho = 0.03, *P* < 0.001), and a trend toward nominal significance in age-bins 8 (*P* = 0.07) and 10 (*P* = 0.05).

### Validation in Braincloud data

Filtered Braincloud data contained dorsolateral prefrontal cortex expression information on 14,773 autosomal genes from 112 subjects (75 males). In covariate-corrected data, expression similarity is dependent on expression variance. Therefore, we compared the standard deviation of expression across all genes overlapping with BrainSpan exon microarray data. We found these estimates to be strongly correlated across datasets (rho = 0.40, *P* < 2.2 × 10^−16^, Spearman correlation), suggesting that preprocessing resulted in high cross-dataset comparability. Since the Braincloud data contained no subjects in age groups 1 and 2 (i.e., age-bin 1 only consisted of subjects in age group 3), age-bin 1 was not used for further analysis. Assessment of expression similarity differences using BrainSpan exon microarray PFC-MSC variability genes validated the decreased similarity in males (*P* < 0.001, Supplementary Fig. 5), which was less pronounced in Braincloud data and driven by genes from age-bins 8 and 9. Consistent with BrainSpan results, co-expression with schizophrenia susceptibility genes was significant in age-bin 2 (rho = 0.03, *P* < 0.001), age-bin 9 (rho = 0.07, *P* < 0.001), and age-bin 10 (rho = 0.03, *P* < 0.001) and showed a trend toward significance in age-bin 8 (rho = 0.01, *P* = 0.08).

## Discussion

The present results demonstrate that the similarity of gene expression profiles in males shows a brain region-specific decrease compared to females. Some of the genes driving this effect were co-expressed with schizophrenia susceptibility genes, in a regionally specific and age-dependent manner. Importantly, co-expression was found in the brain regional cluster encompassing the prefrontal cortex during fetal brain development, confirming a core prediction of the neurodevelopmental hypothesis of schizophrenia^6^. Additionally, and again as predicted by this hypothesis, significant co-expression was further found during adolescence. Similar differences of expression similarity were found in RNAseq data acquired on a subset of the same samples. In this dataset, we further replicated associations between variability and susceptibility genes in data from adolescent donors, but found no associations during the fetal period. Expression similarity differences were further validated in the independent Braincloud data and significant co-expression was found in samples from fetal, as well as adolescent donors.

Co-expression did not depend on how genes were selected from a given susceptibility locus and exceeded that observed for major depression (in age-bin 1 by a small margin) and coronary artery disease in the early fetal phase, as well as during adolescence. We observed that genes selected from randomly chosen loci stratified for DNA sequence variability showed a broadly similar, although less pronounced, co-expression trend compared to schizophrenia genes. In contrast, genes selected randomly without consideration of DNA sequence variability were not co-expressed with variability genes, on average. This may suggest that sequence variability associated with schizophrenia susceptibility loci impacted on diversification of gene expression and the sex differences observed in the present study.

Genes from the fetal and adolescent periods were involved in synaptic processes, which have been implicated in schizophrenia by a range of genetic, histopathological, neuroimaging, pharmacological, and neurotransmitter studies^40–44^. They are affected by genetic and environmental risk in particular during early life, leading to subsequent impairments in synaptic plasticity and connectivity^45^. The lack of overlap between variability genes from the fetal period and adolescence may hint at biologically divergent risk processes that converge on the same synaptic pathways.

The main limitation of the present study is sample size. The primary analysis of the BrainSpan data reported that brain region and age are stronger modulators of gene expression compared to sex or inter-individual variation^19^. Therefore, the present study focused on analyses that are stratified by regional clusters and age-bins, with significant impact on sample numbers available for a given analysis. In the BrainSpan dataset, data from multiple brain regions was available for most donors. We performed a donor-wise bootstrapping procedure during all resampling analyses, to account for the non-independence of the samples. This procedure further accounted for potential effects arising from differences in donor numbers between sexes, further reducing the effective sample size. The low donor number per regional cluster age-bin combination prevented meaningful permutation of gender. Therefore, random “variability genes” were created by randomly sampling genes, stratified by expression variance. This may have led to bias, due to the potential correlation among the actual variability genes that is not captured by the procedure employed here. The low sample number in all three investigated datasets also limits the power to identify and validate significant associations, including expression similarity differences and co-expression between variability and susceptibility genes. This may have contributed to the partial non-replication of findings across datasets.

Another limitation is that we selected a single susceptibility gene per locus to prevent statistical bias, but this selection may not accurately reflect genetic schizophrenia risk. By comparison, other studies have previously selected susceptibility genes by extracting all genes within a given locus^5^ or by focusing on effectors of index variant eQTLs^35^. Another interesting aspect is that the present findings may relate to underlying, variable phenotypes, such as personality traits and comorbid psychiatric conditions. Furthermore, we aimed to account for the effects of known and unknown confounders during all analyses, but this may not have comprehensively captured experimental artefacts that may have influenced between-subject or gene–gene correlations. Finally, we did not use genetic association data to correct for potential subject relatedness or population structure, due to data availability and sample size limitations.

In conclusion, this study indicates sex-specific genetic mechanisms operating during fetal brain development linked to the variability of prefrontal brain gene expression during adolescence, as predicted by the neurodevelopmental hypothesis of schizophrenia. These effects may contribute to the well-established clinical sex differences of schizophrenia and underlying gene sets may be valuable for biologically stratified exploration of the illness’s etiology.

## Electronic supplementary material

Supplementary Figures

Supplementary Tables

Supplementary Dataset
