# Supplementary material for: Male increase in brain gene expression variability is linked to genetic risk for schizophrenia
Source: Transl Psychiatry. 2018 Aug 1;8:140. doi: 10.1038/s41398-018-0200-0 (PMC6070530; doi:10.1038/s41398-018-0200-0)
Supplement: Supplementary file 1 — Supplementary Figures [file 41398_2018_200_MOESM1_ESM.docx]

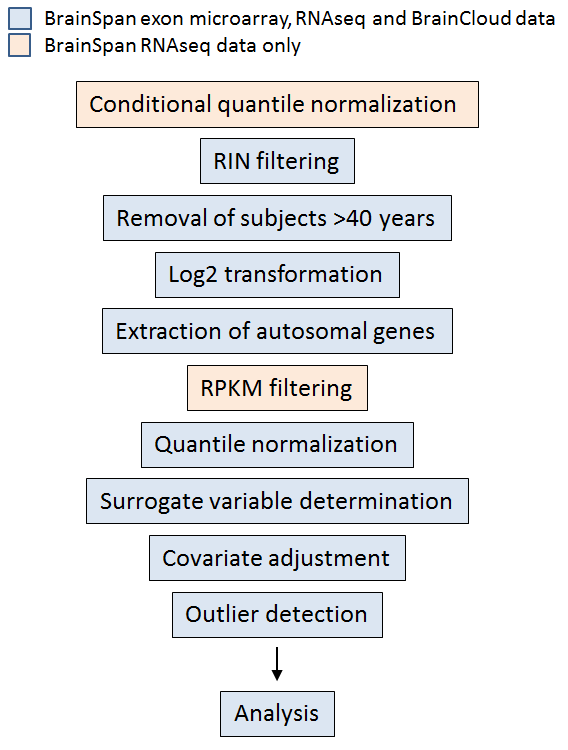


**Supplementary Figure 1. Schematic overview of the preprocessing steps performed for the microarray and RNAseq data used in the present study.**


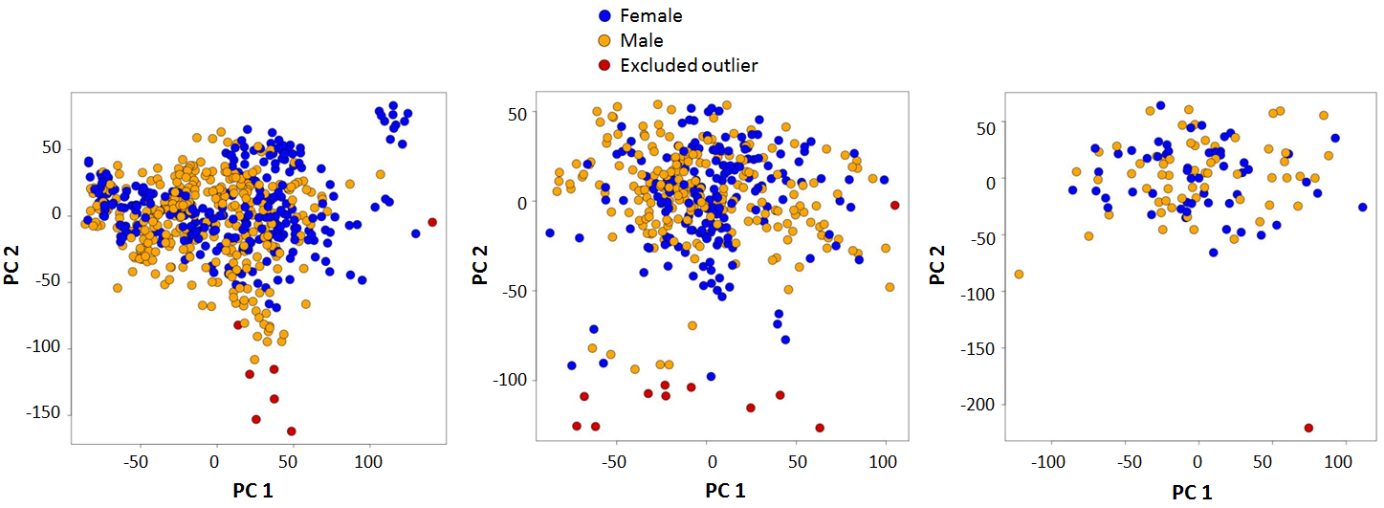


**Supplementary Figure 2. PCA scores plots of BrainSpan exon microarray (left panel), BrainSpan RNAseq (middle panel) and Braincloud (right panel) data.** Excluded outliers are shown in red.


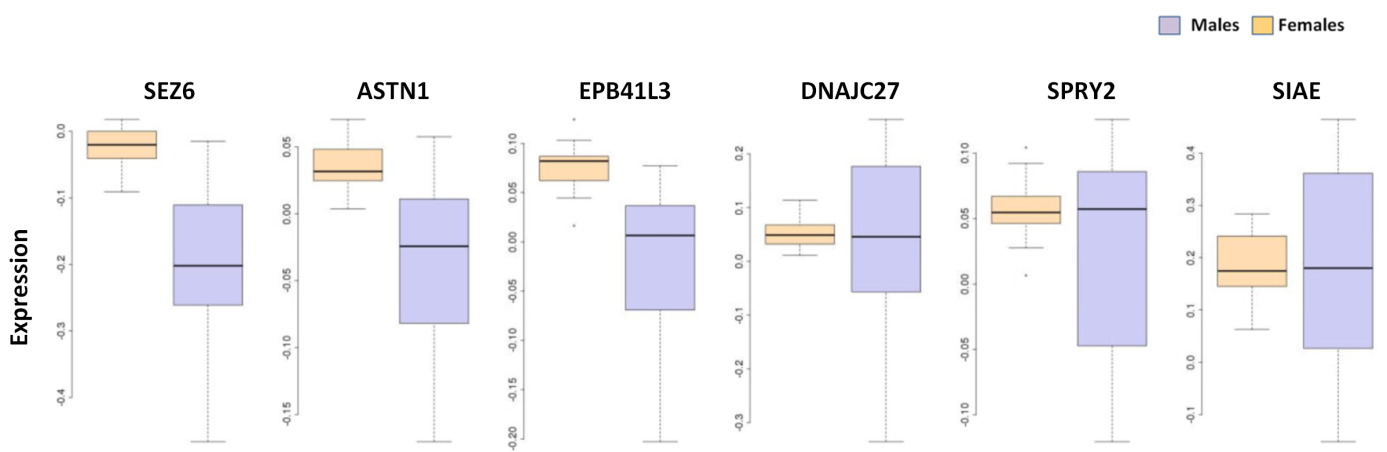


**Supplementary Figure 3. Examples of variability genes in the PFC-MSC cluster, age bin 9**. The three panels on the left show the three genes with the most significant expression difference between males and females. The three panels on the right the genes with the least significant sex-difference in expression. Significance was determined using Wilcoxon rank-sum tests, which do, however, not account for the non-independence between the multiple samples from a given donor.

**
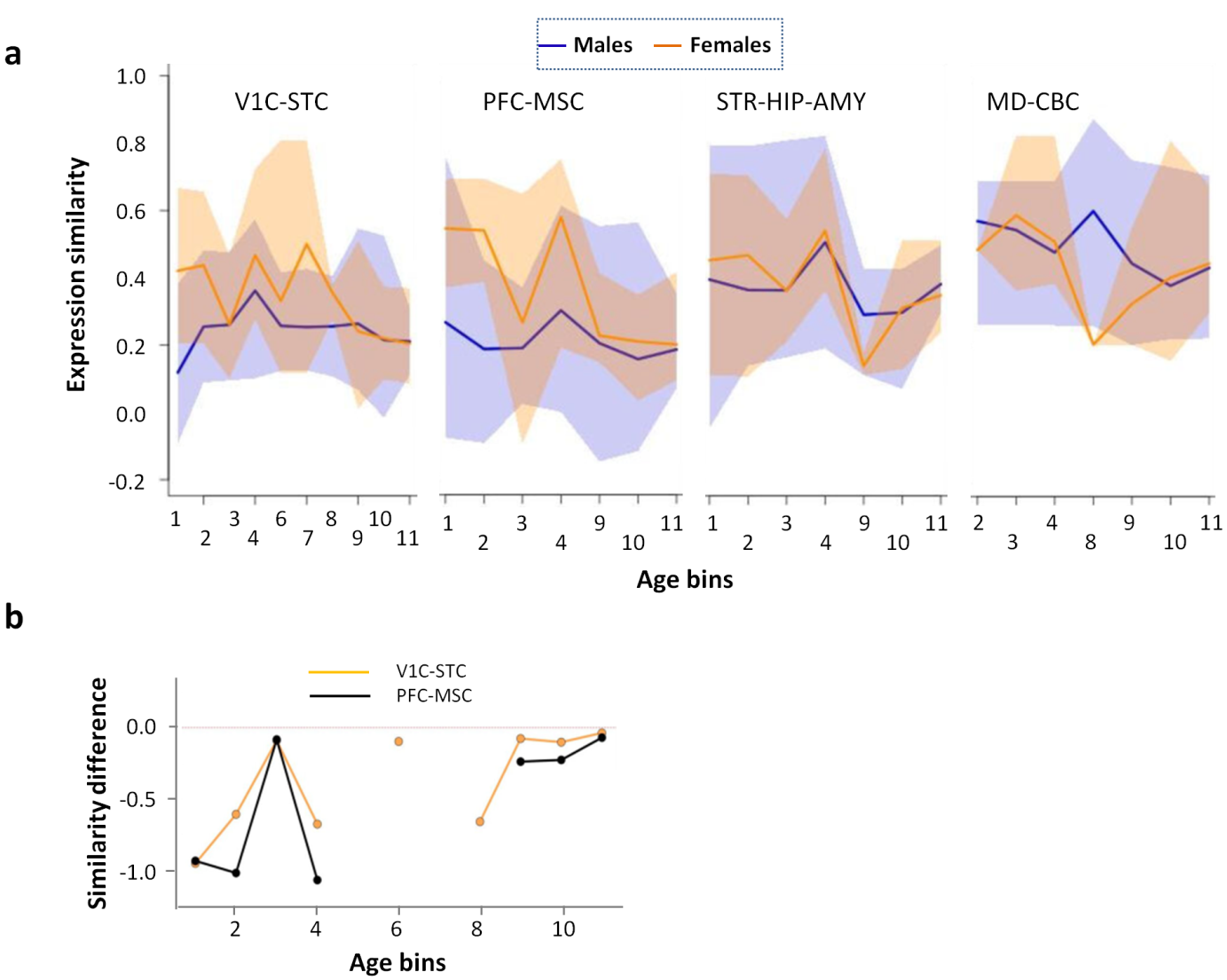
**

**Supplementary Figure 4. Sex differences in expression similarity in BrainSpan RNAseq data. a)** Expression similarity for four brain regional clusters: V1C-STC, PFC-MSC, STR-HIP-AMY, and MD-CBC for males (blue) and females (orange). The panels display mean estimates (solid lines) and 95% confidence intervals (shaded areas). **b)** Differences of expression similarity (male coexpression – female coexpression) for BrainSpan exon microarray ‘variability genes’, assessed separately for each given age bin in BrainSpan RNAseq data.


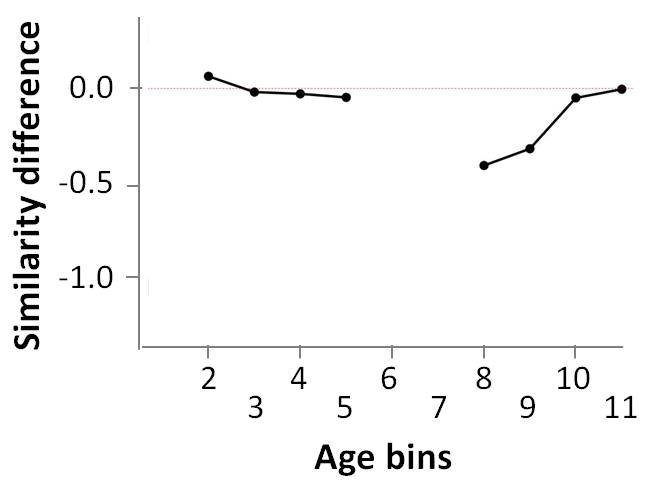


**Supplementary Figure 5.** Differences of expression similarity (male coexpression – female coexpression) for BrainSpan exon microarray ‘variability genes’, assessed separately for each given age bin in Braincloud data.
