# Supplementary material for: Male increase in brain gene expression variability is linked to genetic risk for schizophrenia
Source: Transl Psychiatry. 2018 Aug 1;8:140. doi: 10.1038/s41398-018-0200-0 (PMC6070530; doi:10.1038/s41398-018-0200-0)
Supplement: Supplementary file 2 — Supplementary Tables [file 41398_2018_200_MOESM2_ESM.docx]

**Supplementary Table 1:** Brain regions and regional clusters used in the present study.

| **Cluster** | **Region** | **Description** |
| --- | --- | --- |
| 1 | V1C | primary visual cortex |
| 1 | ITC | inferior temporal cortex |
| 1 | IPC | posterior inferior parietal cortex |
| 1 | A1C | primary auditory cortex |
| 1 | STC | superior temporal cortex |
|  |  |  |
| 2 | M1C | primary motor cortex |
| 2 | S1C | primary somatosensory cortex |
| 2 | VFC | ventral prefrontal cortex |
| 2 | MFC | medial prefrontal cortex |
| 2 | DFC | dorsal prefrontal cortex |
| 2 | OFC | orbital prefrontal cortex |
|  |  |  |
| 3 | STR | striatum |
| 3 | HIP | hippocampal anlage (periods 1–2), hippocampus (periods 3–13) |
| 3 | AMY | amygdala |
|  |  |  |
| 4 | MD | mediodorsal nucleus of the thalamus |
| 4 | CBC | cerebellar cortex |

**Supplementary Table 2:** Grouping of subjects into age bins as performed by Willsey et al ^1^. Periods of human brain development were taken from Kang et al ^2^.

| **Age bin** | **Period** | **Ages** | **Description** |
| --- | --- | --- | --- |
| 1 | 1,2,3 | 6 PCW – 13 PCW | embryonic to early fetal |
| 2 | 2,3,4 | 9 PCW – 16 PCW | early fetal to early mid-fetal |
| 3 | 3,4,5 | 12 PCW – 19 PCW | early fetal to early mid-fetal |
| 4 | 4,5,6 | 16 PCW – 22 PCW | early mid-fetal to late mid-fetal |
| 5 | 5,6,7 | 17 PCW – 37 PCW | early mid-fetal to late fetal |
| 6 | 6,7,8 | 21 PCW – 6 months | late mid-fetal to neonatal & early infancy |
| 7 | 7,8,9 | 25 PCW – 1 year | Late fetal to late infancy |
| 8 | 8,9,10 | 4 months – 4 years | neonatal & early infancy to early childhood |
| 9 | 9,10,11 | 10 months – 11 years | late infancy to middle and late childhood |
| 10 | 10,11,12 | 2 years – 19 years | early childhood to adolescence |
| 11 | 11,12,13 | 11 years – 40 years | Adolescence to young adulthood |

**Supplementary Table 3:** Schizophrenia susceptibility genes used in the present study. Susceptibility loci were taken from a study by the *Schizophrenia Working group of the Psychiatric Genomics Consortium* ^3^. Rank: rank of significance of case-control difference described in ^3^. Chr and position: chromosomal position of index SNP. Gene: gene in closest chromosomal position to the index SNP of a given locus. If a given locus contained multiple index SNPs (annealed locus), the gene in closest proximity to the most significant index SNP was chosen. Genes marked with # were not annotated by the R library org.Hs.eg.db. Genes marked with an asterisk were not part of the BrainSpan data.

| **Rank** | **Index SNP** | **Chr** | **Position** | **Gene** |
| --- | --- | --- | --- | --- |
| 1 | rs115329265 | MHC locus position | | |
| 2 | rs1702294 | 1 | 98501984 | MIR137* |
| 3 | rs11191419 | 10 | 104612335 | C10orf32^#^ |
| 4 | rs2007044 | 12 | 2344960 | CACNA1C |
| 5 | rs4129585 | 8 | 143312933 | TSNARE1 |
| 6 | rs35518360 | 4 | 103146890 | SLC39A8 |
| 7 | chr7_2025096_I | 7 | 2025096 | MAD1L1 |
| 8 | rs4391122 | 5 | 60598543 | ZSWIM6* |
| 9 | rs2851447 | 12 | 123665113 | MPHOSPH9 |
| 10 | chr2_200825237_I | 2 | 200825237 | C2orf47 |
| 11 | rs4702 | 15 | 91426560 | FURIN |
| 12 | rs75968099 | 3 | 36858583 | TRANK1* |
| 13 | chr10_104957618_I | Annealed with rs11191419 | | |
| 14 | rs12887734 | 14 | 104046834 | APOPT1* |
| 15 | rs8042374 | 15 | 78908032 | CHRNA3 |
| 16 | rs13240464 | 7 | 110898915 | IMMP2L |
| 17 | rs10791097 | 11 | 130718630 | SNX19 |
| 18 | rs11693094 | 2 | 185601420 | ZNF804A |
| 19 | rs1378559 | X | 21380266 | CNKSR2 |
| 20 | rs7893279 | 10 | 18745105 | CACNB2 |
| 21 | rs12826178 | 12 | 57622371 | SHMT2 |
| 22 | rs12129573 | 1 | 73768366 | LRRIQ3 |
| 23 | rs6704768 | 2 | 233592501 | GIGYF2 |
| 24 | rs55661361 | 11 | 124613957 | NRGN |
| 25 | rs9636107 | 18 | 53200117 | TCF4 |
| 26 | chr11_46350213_D | 11 | 46350213 | DGKZ |
| 27 | rs7907645 | Annealed with rs11191419 | | |
| 28 | chr3_180594593_I | 3 | 180594593 | FXR1 |
| 29 | rs6065094 | 20 | 37453194 | PPP1R16B |
| 30 | rs11682175 | 2 | 57987593 | VRK2 |
| 31 | rs950169 | 15 | 84706461 | ADAMTSL3 |
| 32 | rs72934570 | 18 | 53533189 | TCF4 |
| 33 | rs6434928 | 2 | 198304577 | SF3B1 |
| 34 | rs9607782 | 22 | 41587556 | EP300 |
| 35 | rs36068923 | 8 | 111485761 | KCNV1 |
| 36 | rs17194490 | 3 | 2547786 | CNTN4 |
| 37 | rs2514218 | 11 | 113392994 | DRD2 |
| 38 | rs75059851 | 11 | 133822569 | IGSF9B |
| 39 | rs2535627 | 3 | 52845105 | ITIH4 |
| 40 | rs12691307 | 16 | 29939877 | KCTD13 |
| 41 | chr22_39987017_D | 22 | 39987017 | CACNA1I |
| 42 | rs7432375 | 3 | 136288405 | STAG1 |
| 43 | chr18_52749216_D | Annealed with rs9636107 | | |
| 44 | rs111294930 | 5 | 152177121 | GRIA1 |
| 45 | rs2973155 | Annealed with rs111294930 | | |
| 46 | rs5937157 | X | 68377126 | PJA1 |
| 47 | rs4523957 | 17 | 2208899 | SRR |
| 48 | rs12704290 | 7 | 86427626 | GRM3 |
| 49 | rs12903146 | 15 | 61854663 | VPS14C^#^ |
| 50 | rs11210892 | 1 | 44100084 | PTPRF |
| 51 | rs2905426 | 19 | 19478022 | MAU2* |
| 52 | rs140505938 | 1 | 150031490 | VPS45 |
| 53 | chr6_84280274_D | 6 | 84280274 | SNAP91 |
| 54 | rs4648845 | 1 | 2387101 | PLCH2 |
| 55 | rs7405404 | 16 | 13749859 | ERCC4 |
| 56 | rs6466055 | 7 | 104929064 | SRPK2 |
| 57 | chr1_8424984_D | 1 | 8424984 | RERE |
| 58 | rs4766428 | 12 | 110723245 | ATP2A2 |
| 59 | rs10520163 | 4 | 170626552 | CLCN3 |
| 60 | rs117074560 | 6 | 96459651 | FUT9 |
| 61 | rs6002655 | 22 | 42603814 | TCF20 |
| 62 | chr2_146436222_I | No gene in proximity | |  |
| 63 | rs9420 | 11 | 57510294 | C11orf31 |
| 64 | rs11027857 | 11 | 24403620 | LUZP2 |
| 65 | rs1498232 | No gene in proximity | |  |
| 66 | rs3735025 | 7 | 137074844 | DGKI |
| 67 | rs11139497 | 9 | 84739941 | TLE1 |
| 68 | rs77149735 | 1 | 243555105 | SDCCAG8 |
| 69 | rs56205728 | 15 | 40567237 | PAK6 |
| 70 | rs2053079 | 19 | 30987423 | ZNF536 |
| 71 | rs16867576 | 5 | 88746331 | MEF2C |
| 72 | rs4330281 | 3 | 17859366 | TBC1D5 |
| 73 | rs3849046 | 5 | 137851192 | ETF1 |
| 74 | rs2693698 | 14 | 99719219 | BCL11B |
| 75 | rs2332700 | 14 | 72417326 | RGS6 |
| 76 | rs1501357 | 5 | 45364875 | HCN1 |
| 77 | rs6984242 | 8 | 60700469 | CA8 |
| 78 | chr1_243881945_I | Annealed with rs77149735 | | |
| 79 | rs79212538 | Annealed with rs111294930 | | |
| 80 | rs3768644 | 2 | 72361505 | CYP26B1 |
| 81 | rs77502336 | 11 | 123394636 | GRAMD1B |
| 82 | rs6704641 | 2 | 200164252 | SATB2 |
| 83 | rs59979824 | 2 | 193848340 | PCGEM1* |
| 84 | rs1106568 | 4 | 176861301 | GPM6A |
| 85 | rs10503253 | 8 | 4180844 | CSMD1 |
| 86 | rs10043984 | Annealed with rs3849046 | | |
| 87 | rs11685299 | 2 | 225391296 | CUL3 |
| 88 | rs7819570 | 8 | 89588626 | MMP16 |
| 89 | rs715170 | Annealed with rs72934570 | | |
| 90 | rs9922678 | 16 | 9946319 | GRIN2A |
| 91 | rs78322266 | Annealed with rs9636107 | | |
| 92 | rs2068012 | 14 | 30190316 | PRKD1 |
| 93 | rs832187 | 3 | 63833050 | C3orf49 |
| 94 | rs8044995 | 16 | 68189340 | NFATC3 |
| 95 | chr2_149429178_D | 2 | 149429178 | EPC2 |
| 96 | rs8082590 | 17 | 17958402 | GID4* |
| 97 | rs12148337 | 15 | 70589272 | TLE3 |
| 98 | rs12325245 | 16 | 58681393 | CNOT1 |
| 99 | rs2239063 | Annealed with rs2007044 | | |
| 100 | rs12522290 | Annealed with rs111294930 | | |
| 101 | rs10803138 | Annealed with rs77149735 | | |
| 102 | rs73229090 | 8 | 27442127 | CLU |
| 103 | rs324017 | Annealed with rs12826178 | | |
| 104 | rs12845396 | X | 6029533 | NLGN4X |
| 105 | rs55833108 | Annealed with rs11191419 | | |
| 106 | rs9841616 | Annealed with chr3_180594593_I | | |
| 107 | rs76869799 | Annealed with rs1702294 | | |
| 108 | rs1339227 | 6 | 73155701 | RIMS1 |
| 109 | chr7_24747494_D | 7 | 24747494 | DFNA5 |
| 110 | rs4388249 | 5 | 109036066 | MAN2A1 |
| 111 | rs215411 | 4 | 23423603 | MIR548AJ2* |
| 112 | rs11740474 | 5 | 153680747 | GALNT10 |
| 113 | rs1023500 | Annealed with rs6002655 | | |
| 114 | rs12421382 | 11 | 109378071 | C11orf87 |
| 115 | rs211829 | 7 | 110048893 | IMMP2L |
| 116 | rs679087 | 12 | 29917265 | TMTC1 |
| 117 | rs75575209 | Annealed with rs11682175 | | |
| 118 | rs7801375 | 7 | 131567263 | PODXL |
| 119 | rs14403 | Annealed with rs77149735 | | |
| 120 | rs6670165 | 1 | 177280121 | BRINP2* |
| 121 | rs7523273 | 1 | 207977083 | CD46 |
| 122 | rs7267348 | 20 | 48131036 | PTGIS |
| 123 | rs4240748 | 12 | 92246786 | C12orf79^#^ |
| 124 | rs2909457 | 2 | 162845855 | DPP4 |
| 125 | rs56873913 | 19 | 50091199 | PRRG2 |
| 126 | rs190065944 | Annealed with rs8042374 | | |
| 127 | rs10860964 | 12 | 103596455 | C12orf42 |
| 128 | chr5_140143664_I | 5 | 140143664 | PCDHA1* |

**Supplementary Table 4:** Ontological terms associated with heterogeneity genes of age bins 8, 9 and 10 in the PFC-MSC cluster, as determined using the DAVID tool ^4^. P-values were corrected for the False Discovery Rate (FDR) according to the method of Benjamini and Hochberg.

| **Category** | **P(FDR)** |
| --- | --- |
| calcium ion binding | 2.60E-04 |
| Synapse | 3.50E-03 |
| Epilepsy | 3.80E-03 |
| Phosphoprotein | 4.00E-03 |
| Ion channel | 4.20E-03 |
| Parkinson disease | 5.10E-03 |
| Membrane | 5.90E-03 |
| Alternative splicing | 1.10E-02 |
| Glycoprotein | 2.50E-02 |
| Ion transport | 2.80E-02 |
| Parkinsonism | 3.20E-02 |
| Cell membrane | 3.40E-02 |
| Cell adhesion | 3.40E-02 |
| Cell junction | 4.10E-02 |
| plasma membrane | 4.90E-02 |

**Supplementary** **Table 5:** Ontological terms associated with heterogeneity genes of age bins 1 and 2 in the PFC-MSC cluster, as determined using the DAVID tool ^4^. P-values were corrected for the False Discovery Rate (FDR) according to the method of Benjamini and Hochberg.

| **Category** | **P(FDR)** |
| --- | --- |
| Synapse | 3.30E-03 |
| Postsynaptic cell membrane | 5.50E-03 |
| dendrite | 1.90E-02 |
| cell junction | 2.20E-02 |
| Cell membrane | 2.60E-02 |
| postsynaptic membrane | 3.50E-02 |
| Membrane | 4.50E-02 |
| chemical synaptic transmission | 4.60E-02 |

**Supplementary** **Table 6:** Ontological terms associated with heterogeneity genes of age bins 1 and 2 in the PFC-MSC cluster, as determined using the DAVID tool ^4^. P-values were corrected for the False Discovery Rate (FDR) according to the method of Benjamini and Hochberg.

| **Category** | **P(FDR)** |
| --- | --- |
| Phosphoprotein | 1.60E-07 |
| CARM1 and Regulation of the Estrogen Receptor | 7.70E-03 |
| Cell division and chromosome partitioning | 4.40E-02 |

**Supplementary** **Table 7:** Age and sex distribution for donors part of the age-windowing analysis. Age is shown as mean ± sd.

| **Age-window** | **age** | **sex (m/f)** |
| --- | --- | --- |
| 1 | 1.9 ± 1.3 | 28/9 |
| 2 | 4.8 ± 3.1 | 20/9 |
| 3 | 6.8 ± 3.7 | 15/15 |
| 4 | 9.2 ± 3.5 | 15/15 |
| 5 | 10.9 ± 3.7 | 21/12 |
| 6 | 13.3 ± 3.9 | 16/18 |

**Supplementary Table 8:** BrainSpan RNAseq sample numbers for males and females across 11 age bins and 4 brain regional clusters after data preprocessing (1: V1C-STC, 2: PFC-MSC, 3: STR-HIP-AMY, 4: MD-CBC, see Supplementary Table 1 for details). Subject numbers are shown in brackets.

|  |  | | **Males** | | | | | **Females** | | | | |
| --- | --- | --- | --- | --- | --- | --- | --- | --- | --- | --- | --- | --- |
| Age bin | Regional cluster | **1** | | **2** | **3** | **4** | **1** | | **2** | **3** | **4** |  |
| 1 |  | 10 (3) | | 21 (4) | 10 (4) | 0 (0) | 19 (4) | | 23 (4) | 12 (4) | 2 (2) |  |
| 2 |  | 23 (5) | | 29 (6) | 15 (6) | 4 (3) | 19 (4) | | 23 (4) | 12 (4) | 2 (2) |  |
| 3 |  | 23 (5) | | 25 (5) | 13 (5) | 4 (3) | 27 (6) | | 32 (6) | 17 (6) | 5 (4) |  |
| 4 |  | 18 (4) | | 19 (4) | 10 (4) | 5 (4) | 9 (3) | | 9 (2) | 5 (2) | 4 (3) |  |
| 5 |  | 4 (1) | | 6 (1) | 3 (1) | 1 (1) | 12 (5) | | 10 (3) | 5 (2) | 4 (3) |  |
| 6 |  | 12 (3) | | 13 (3) | 9 (3) | 5 (3) | 4 (2) | | 1 (1) | 0 (0) | 1 (1) |  |
| 7 |  | 12 (3) | | 11 (3) | 6 (2) | 5 (3) | 3 (1) | | 1 (1) | 0 (0) | 0 (0) |  |
| 8 |  | 13 (4) | | 15 (5) | 8 (3) | 6 (4) | 5 (2) | | 2 (1) | 1 (1) | 2 (2) |  |
| 9 |  | 15 (4) | | 17 (5) | 6 (3) | 4 (4) | 10 (3) | | 8 (2) | 3 (2) | 3 (3) |  |
| 10 |  | 19 (5) | | 17 (5) | 8 (5) | 4 (4) | 20 (5) | | 20 (4) | 9 (4) | 6 (5) |  |
| 11 |  | 28 (7) | | 30 (6) | 13 (7) | 8 (6) | 30 (6) | | 35 (6) | 16 (6) | 8 (6) |  |

**Supplementary Table 9:** BrainCloud subject numbers for each age-bin after data preprocessing.

| Age bin |  | **Males** | **Females** |
| --- | --- | --- | --- |
| 1 |  | 2 | 2 |
| 2 |  | 8 | 5 |
| 3 |  | 19 | 19 |
| 4 |  | 17 | 17 |
| 5 |  | 11 | 14 |
| 6 |  | 6 | 1 |
| 7 |  | 6 | 1 |
| 8 |  | 10 | 4 |
| 9 |  | 4 | 4 |
| 10 |  | 30 | 14 |
| 11 |  | 46 | 14 |

**References**

1. Willsey, A.J.*, et al.* Coexpression networks implicate human midfetal deep cortical projection neurons in the pathogenesis of autism. *Cell* **155**, 997-1007 (2013).

2. Kang, H.J.*, et al.* Spatio-temporal transcriptome of the human brain. *Nature* **478**, 483-489 (2011).

3. Schizophrenia Working Group of the Psychiatric Genomics, C. Biological insights from 108 schizophrenia-associated genetic loci. *Nature* **511**, 421-427 (2014).

4. Huang da, W., Sherman, B.T. & Lempicki, R.A. Systematic and integrative analysis of large gene lists using DAVID bioinformatics resources. *Nat Protoc* **4**, 44-57 (2009).
